# The role of premenstrual syndrome in hedonic hunger and food craving during the menstrual cycle

**DOI:** 10.1017/jns.2025.10038

**Published:** 2025-09-19

**Authors:** Ebru Candan, Ziya Erokay Metin, M. Merve Tengilimoglu-Metin

**Affiliations:** 1 Department of Nutrition and Dietetics, Faculty of Health Sciences, Hacettepe University, Sihhiye, Ankara, Turkey; 2 Department of Nutrition and Dietetics, Gulhane Faculty of Health Sciences, University of Health Sciences, Ankara, Turkey

**Keywords:** Appetite, food craving, hedonic hunger, premenstrual syndrome, PMS, Premenstrual Syndrome, PFS, The Power of Food Scale, FCQ-T, Food Craving Questionnaire-Trait, BMI, Body Mass Index, PSS, Premenstrual Syndrome Scale, GLP-1, Glucagon-like Peptide 1, CCK, Cholecystokinin

## Abstract

Differences in appetite, food intake, eating behaviours, and food preferences can occur throughout the menstrual cycle. Premenstrual syndrome (PMS) is associated with significant emotional and physiological changes, including altered appetite and food cravings. Therefore, the relationship between PMS and hedonic hunger, food craving of individuals during the menstrual cycle phases were investigated in this study. This study was conducted on 150 women volunteers. Research data were collected using a web-based questionnaire. Four assessment stages were scheduled for each woman, and they were classified in phases according to the onset of menstruation. Participants completed premenstrual syndrome scale and anthropometric measurements were taken based on their statements at the initial assessment stage. In the first, second, and third phases of menstrual cycle, a questionnaire form including the power of food scale (PFS) and Food Craving Questionnaire-Trait (FCQ-T) were applied.

The average age, age of menarche, menstrual cycle length, and bleeding time of the participants were 22.0 ± 2.0, 13 ± 1, 27.7 ± 3, 5.9 ± 1.3, respectively. Women with PMS showed significantly higher total PFS scores compared to those without PMS during the luteal phase (mean ± SD: 3.5 ± 0.6 vs. 2.9 ± 0.7, *p* < 0.01). Energy intake also increased significantly in the PMS group during this phase (mean ± SD: 2,200 ± 310 kcal/day vs. 1,880 ± 290 kcal/day, *p* < 0.01). The PFS total scores of participants in phase 1 and phase 2 differ significantly according to BMI classification (*p* = 0.017; *p* = 0.013). According to the presence of PMS, phase 1, phase 2, and phase 3, PFS total and sub-factor scores of women differ significantly (*p* < 0.05). The scores of those with PMS were higher than the scores of those without PMS. In conclusion, the presence of PMS affects hedonic hunger during the menstrual cycle phases.

## Graphical abstract

**Figure:**
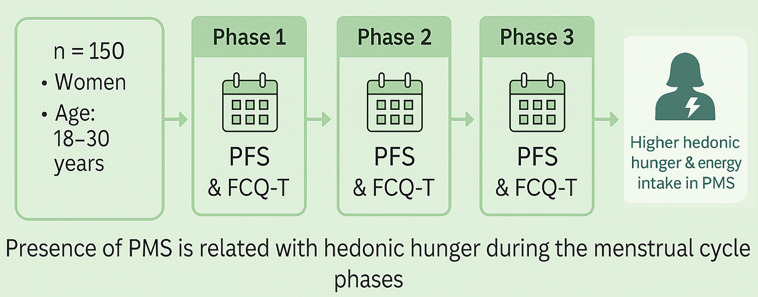

## Introduction

Premenstrual symptoms have a negative impact on quality of life due to their physiological effects.^(1)^ Premenstrual syndrome (PMS) is a common health problem in women of reproductive age and can cause negative conditions such as appetite disturbances.^(2,3)^ PMS is characterized by a constellation of recurring physical, emotional, cognitive, and behavioural symptoms that occur during the luteal phase of the menstrual cycle and typically resolve with the onset of menstruation.^(4)^ According to Yonkers et al., PMS is diagnosed when these symptoms are sufficiently severe to interfere with daily functioning, and their cyclical pattern is confirmed prospectively across at least two menstrual cycles.^(5)^ Among the behavioural changes reported in women with PMS, alterations in appetite and food cravings are particularly common. Previous studies have shown that women with PMS often experience increased cravings for sweets and carbohydrate-rich foods, heightened hedonic drive to eat, and more frequent episodes of emotional eating during the luteal phase;^(2)^ however, the relationship with hedonic hunger is not clear.^(6,7)^ It is conceivable that fluctuations in ovarian hormone levels during the stages of the menstrual cycle have an effect on appetite and so hedonic hunger.^(8,9)^

Hedonic hunger is defined as the desire to consume food when there is no physical need.^(10)^ The power of food scale (PFS) was developed to measure hedonic hunger and to relate it to other parameters.^(11)^ The PFS has been used in many studies since its publication.^(12,13)^ Previous studies have shown that PFS scores can predict interest in energy-dense and palatable foods.^(14,15)^ Considering that hedonic processes are significantly effective in food consumption and hormonal fluctuations occur during the menstrual cycle, measuring hedonic hunger in women with the food power scale can be considered as an important point.^(16)^ In addition, food craving is commonly reported in menstruating women,^(17)^ and in a recent cross-sectional study, the prevalence of food craving during the menstrual cycle in adult women was 91.78%.^(18)^ Food craving refers to an intense desire for a particular food and is a definition that differs from general hunger and craved food is usually energy-dense and fatty foods.^(19)^ In this context, the food craving questionnaire was developed to measure the frequency and intensity of craving.^(20)^

The menstrual cycle controlled by the endocrine system is a series of physiological changes that can occur in childbearing age women is necessary for reproduction.^(21)^ Many women of reproductive age suffer from PMS. According to a meta-analysis by Direkvand-Moghadam et al., the global pooled prevalence of PMS is approximately 47.8%, highlighting its significant impact on women of reproductive age.^(22)^ Emotional, physical, cognitive, and behavioural symptoms — such as pain, irritability, anxiety, and changes in appetite — may occur during the late luteal phase of the menstrual cycle. However, in individuals with PMS, these symptoms are more intense, recurrent, and sufficiently disruptive to daily functioning.^(23)^ Nutrition, stress, and mood changes are environmental factors that can influence the menstrual cycle.^(24)^ The menstrual cycle is divided into phases according to hormonal fluctuations,^(25)^ and many studies have shown that there are significant differences in appetite, food intake, eating habits, and food preferences throughout the menstrual cycle.^(26–28)^ These variations are explained in part by the impact of oestrogen and progesterone on gastric emptying and the secretion of gastrointestinal hormones such as glucagon-like peptide (GLP-1) and cholecystokinin (CCK), which are important factors in regulating appetite and energy intake.^(29)^ Increased oestrogen hormone level in the follicular phase of the menstrual cycle reduces appetite, causing a decrease in the desire to eat.^(30)^ Mood symptoms such as anxiety, irritability, and fatigue during the luteal phase are thought to result from the decline in progesterone and oestrogen levels that occurs just prior to menstruation, particularly in women who are sensitive to hormonal changes.^(5)^ These symptoms have been associated with a decrease in serotonin release, leading to increased consumption of carbohydrate-rich foods, especially simple sugars. These foods enhance the availability of tryptophan — the precursor of serotonin — in the brain, which may help improve mood and emotional regulation.^(25,30)^ Studies have revealed that women prefer foods with high carbohydrate content and sweets more in the luteal phase.^(25,28,31)^ While many studies report that hormonal fluctuations during the menstrual cycle influence appetite and food cravings, other research has found no significant or consistent effects.^(6,32)^ In addition, it has been reported that women with PMS have greater appetite and sweet food consumption in the late luteal period than women without PMS.^(33)^ The relationship between nutrient intake and hormonal fluctuations during the menstrual cycle is complicated and includes several regulatory systems, although the exact mechanisms are not fully understood.^(29)^ In some phases of the menstrual cycle (particularly the luteal phase), increased food cravings and poor eating habits may cause an increase in body weight and some nutrient deficiencies and may increase the risk of lifestyle-related diseases (diabetes and hypertension).^(30)^ Additionally, body mass index (BMI) has been shown to influence both physiological hunger and hedonic eating behaviour, with individuals classified as overweight or obese often exhibiting greater responsiveness to food cues and cravings compared to those with normal weight.^(34,35)^ Therefore, this study aimed to investigate whether hedonic hunger, food craving, energy intake, and nutrient intake could be modified during three different phases of the menstrual cycle (follicular, ovulatory, and late luteal phases) in women with varying BMI. Also, the purpose of this study is to gain new insights into individual differences in susceptibility to changes in appetite across the menstrual cycle and between those with and without PMS.

## Methods

### Participants

This study was conducted on 150 women volunteers’ ages of 19–24 who were regularly menstruating included. Regularly menstruating was defined as having a self-reported cycle length between 24 and 35 days, with consistent timing (±3 days) over the previous six months.^(36)^ An invitation was sent to participants via social media (Facebook, Instagram, LinkedIn, Twitter, and WhatsApp) using the snowball sampling method. Women were excluded if they were using regularly any drugs (birth control pills, antidepressants, antihypertensive, antimicrobials, analgesics, etc.), chronic, or systemic diseases, or psychiatric diseases or diagnosed eating disorders; receiving eating behaviour therapy; and being pregnant or lactating. The Hacettepe University Non-Interventional Clinical Researches Ethics Board approved the protocol (approval number: GO 21/908), conducted in accordance with Declaration of Helsinki. All participants gave written consent after receiving verbal and written information.

### Study design

Each woman was followed for a full menstrual cycle (28 to 30 days, based on self-reported history), with prior cycle length information and data collected via a structured questionnaire administered through Google Forms. Four assessment stages were scheduled for each woman and all women were not tested in the same order (phases 1, 2, 3). Women answered premenstrual syndrome scale (PSS) and anthropometric measurements (height (cm) and weight (kg)) were taken based on their statements at the initial assessment stage. Body mass index (BMI) was calculated using self-reported weight and height (kg/m^2^). Participants were grouped based on WHO criteria: normal weight (BMI 18.5–24.9), overweight (BMI 25.0–29.9), and obese (BMI ≥30.0).^(37)^ These categories were used for subgroup analyses of hedonic hunger and food craving. For statistical analysis, participants were grouped into two BMI categories: normal weight (BMI 18.5–24.9) and overweight/obese (BMI ≥25.0), based on WHO classification criteria. This grouping was used to compare hedonic hunger and food craving scores across menstrual cycle phases. Participants reported the first day of their last menstrual period, and cycle phases were estimated based on typical cycle length. In the first, second, and third phases of menstrual cycle a questionnaire form including The PFS, Food Craving Questionnaire-Trait (FCQ-T) were applied.

- Initial assessment: the menstrual cycle stage was learned, and the cycle dates were noted to be followed.
- Second assessment: Phase 1 (Follicular phase): Days 1–5, corresponding to the menstruation period.
- Third assessment: Phase 2 (Ovulatory phase): Days 11–16, representing the mid-cycle ovulation period.
- Fourth assessment: Phase 3 (Luteal phase): Days 21–26, corresponding to the late luteal period, ending one day before the onset of the next menstruation.

### Measures

#### Premenstrual syndrome scale

The PSS is a 44-item measure designed to examine PMS. The questionnaire was developed by Gençdoğan and tested for its validity and reliability in Turkish.^(38)^ The participants rated all items on a five-level scale ranging from never to always. The PSS, in which mean scores ≥ 2,5 are used as a criterion for PMS. Participants scoring below this threshold were considered non-PMS.

#### The power of food scale

The PFS is a 15-item measure questionnaire designed to assess differences in individuals’ hedonic hunger states. Responses are measured on a five-point Likert-type scale ranging from do not agree at all to strongly agree, with higher scores indicating greater psychological impact of the food-based environment. Because the PFS does not include any items that describe the actual food intake, this measure examines appetitive behaviour to food stimuli. The measure is represented by three domain scores and an aggregate score. The domain group scores consist of three factors of “food available”, “food present”, and “food tasted” and were calculated by averaging of each comprised items representing the individual’s responsiveness to the food environment. The aggregate score was calculated as the mean of the three domains. Food available sub-scale assesses general thoughts about food, food present sub-scale assesses attraction to food which is available to the individual and food tasted sub-scale assesses pleasure when food is first tasted. Turkish reliability and validity study of the PFS was conducted by Ulker et al.^(39)^

#### Food craving questionnaire-trait (FCQ-T)

The FCQ-T assess food cravings in nine domains.^(20)^ Participants rated all the items scored on a six-level scale ranging from 1, “never/not applicable”, to 6, “always”. The scores on each sub-domain indicate hedonic psychological drive to palatable foods at different levels of “plans”, “positive reinforcement”, “negative reinforcement”, “loss of control”, “food concern”, “hunger”, “emotions”, “context”, and “guilt”. Turkish reliability and validity study of the PFS was conducted by Akkurt.^(40)^

#### Dietary intake

Dietary intake was assessed using a structured, self-administered 24-hour dietary recall method conducted on one day for each of the three defined menstrual cycle phases. The 24-hour dietary recall form was shared electronically with participants. The form was developed based on structured dietary recall frameworks described in previous research.^(41)^ Participants reported all foods and beverages consumed throughout the day, specifying portion sizes and ingredients. For foods consumed outside the home, standard recipes were used to estimate ingredient quantities. After determining the quantities of consumed items, the nutrient intakes — including energy, macronutrients, and micronutrients — were calculated using the Nutrition Information Systems software (BEBIS), version 6.1.^(42)^

### Statistical analyses

The IBM SPSS statistical package software program, version 23.0, was used for the analysis of all data. Frequency and percentage analyses were used to determine the descriptive characteristics of the employees participating in the research, and average and standard deviation statistics were used in the analysis of the scale. Differences between the ratios of categorical variables in independent groups were analysed with Chi-square and Fisher’s exact tests. Relationships between dimensions determining women’s scale levels were examined through Pearson correlation analyses. Independent groups t-test analyses were used to examine the differences in the scale levels of women according to the two groups. The differentiation status of the scale scores in the phase changes within the BMI groups was analysed with the repeated measurements ANOVA test. Regression analysis was done to predict hedonic hunger.

## Results

According to our data, the average age, age of menarche, menstrual cycle length, and bleeding time of the participants were 22.0 ± 2.0, 13 ± 1, 27.7 ± 3, 5.9 ± 1.3, respectively. The mean age of menarche, menstrual cycle length, and bleeding day of women did not differ according to BMI classification (*p* > 0.05) (Table 1).

**Table 1.** Descriptive characteristics of participants

Variable	Mean ± SD
Age (years)	22.0 ± 2.0
Age at menarche (years)	13.0 ± 1.0
Menstrual cycle length (days)	27.7 ± 3.5
Duration of bleeding (days)	5.9 ± 1.3

Table 2 presents the PFS total and sub-factor scores across menstrual cycle phases by BMI classification. Notably, overweight and obese individuals had significantly higher total PFS scores and sub-factor scores for “food available” and “food present” during phase 1 and phase 2 compared to normal-weight individuals, indicating elevated hedonic hunger in these phases.

**Table 2.** Evaluation of the PFS total and sub-factor scores according to BMI classification and menstrual cycle phases

PFS total score and sub-factors	Menstrual cycle phases	Normal weight (*n* = 75)	Overweight and obese (*n* = 75)	
		Means ± SD	Means ± SD	*p* ^#^
PFS total score	Phase 1	2.8 ± 0.99	3.2 ± 0.92	0.017
	Phase 2	2.8 ± 0.99	3.2 ± 0.90	0.013
	Phase 3	2.9 ± 0.95	3.2 ± 0.96	0.095
p*		0.491	0.853	
Food available	Phase 1	2.5 ± 1.02	2.9 ± 1.07	0.015
	Phase 2	2.4 ± 1.04	2.9 ± 1.00	0.003
	Phase 3	2.6 ± 1.01	2.9 ± 1.12	0.096
p*		0.224	0.786	
Food present	Phase 1	2.9 ± 1.16	3.4 ± 0.93	0.008
	Phase 2	2.9 ± 1.06	3.3 ± 1.04	0.040
	Phase 3	3.0 ± 1.02	3.3 ± 1.03	0.066
p*		0.581	0.274	
Food tasted	Phase 1	3.2 ± 1.13	3.4 ± 1.04	0.146
	Phase 2	3.1 ± 1.13	3.4 ± 1.00	0.110
	Phase 3	3.2 ± 1.06	3.4 ± 1.02	0.268
p*		0.872	0.872	

#Independent group *T*-test *Repeated measures ANOVA test.

Participants’ PFS total score and sub-factor scores of food available, food present, and food tasted did not differ significantly between phases (*p* > 0.05). The PFS total scores of participants in phase 1 and phase 2 differ significantly according to BMI classification (*p* = 0.017; *p* = 0.013). The total PFS score of overweight and obese individuals was found to be higher than those of normal weight. In phase 3, however, the PFS total scores of the participants did not differ significantly according to the BMI classification (*p* > 0.05). Food availability and food present sub-factor scores of participants in phase 1 (*p* = 0.015) and phase 2 (*p* = 0.008) differ significantly according to BMI classification. The food available and food present sub-factor score of overweight and obese individuals was found to be higher than those of normal weight. In phase 3, participants’ food available and food present sub-factor score did not differ significantly according to BMI classification (*p* > 0.05). In phase 1, phase 2, and phase 3, participants’ food tasting sub-factor scores did not differ significantly according to BMI classification (*p* > 0.05) (Table 1).

Table 3 summarizes the Food Craving Questionnaire-Trait (FCQ-T) sub-factor scores across menstrual cycle phases by BMI classification. Significant differences were observed in several sub-factors, particularly in phase 1 and phase 2. Overweight and obese women generally reported higher scores for “plans,” “negative reinforcement,” “loss of control,” and “food concern” in phase 1 compared to normal-weight women. In contrast, some sub-factors such as “plans” and “emotions” showed higher scores in normal-weight women during phase 2 and phase 3. These results suggest that BMI may influence specific aspects of food craving differently across menstrual cycle phases.

**Table 3.** Evaluation of FCQ-T sub-factor scores according to BMI groups and menstrual cycle phases

FCQ-T sub-factors	Menstrual cycle phases	Normal weight (*n* = 75)	Overweight and obese (*n* = 75)	*p* ^#^
		Means ± SD	Means ± SD	
Plans	Phase 1	2.7^b^ ± 1.16	3.2^b^ ± 2.95	0.004
	Phase 2	4.2^a^ ± 1.18	3.7^a^ ± 2.46	0.015
	Phase 3	4.2^a^ ± 1.27	3.8^a^ ± 1.90	0.060
p*		<0.001	0.048	
Positive reinforcement	Phase 1	2.9^b^ ± 1.5	3.3 ± 1.81	0.073
	Phase 2	3.9^a^ ± 1.43	3.6 ± 1.58	0.117
	Phase 3	4.1^a^ ± 1.49	3.8 ± 1.12	0.266
p*		<0.001	0.129	
Negative reinforcement	Phase 1	2.9^b^ ± 1.17	3.5 ± 3.39	0.001
	Phase 2	3.9^a^ ± 1.29	3.4 ± 2.59	0.011
	Phase 3	3.9^a^ ± 1.26	3.5 ± 2.14	0.034
p*		<0.001	0.793	
Loss of control	Phase 1	2.9^b^ ± 1.31	3.5 ± 2.95	0.004
	Phase 2	3.9^a^ ± 1.51	3.4 ± 2.22	0.028
	Phase 3	3.9^a^ ± 1.38	3.6 ± 1.26	0.210
p*		<0.001	0.678	
Food concern	Phase 1	2.6^b^ ± 1.35	3.1^b^ ± 2.28	0.024
	Phase 2	4.3^a^ ± 1.33	3.8^a^ ± 1.75	0.082
	Phase 3	4.3^a^ ± 1.44	4.0^a^ ± 1.05	0.295
p*		<0.001	0.003	
Hunger	Phase 1	2.3^b^ ± 1.29	2.8^b^ ± 2.47	0.015
	Phase 2	4.6^a^ ± 1.29	4.2^a^ ± 1.54	0.126
	Phase 3	4.5^a^ ± 1.43	4.2^a^ ± 1.25	0.214
p*		<0.001	<0.001	
Emotions	Phase 1	3.1^b^ ± 1.26	3.5 ± 1.87	0.064
	Phase2	3.8^a^ ± 1.35	3.3 ± 2.21	0.029
	Phase 3	3.8^a^ ± 1.33	3.3 ± 2.24	0.026
p*		0.004	0.633	
Context	Phase 1	2.5^b^ ± 1.44	3.3 ± 3.29	0.001
	Phase 2	4.3^a^ ± 1.45	3.7 ± 2.73	0.007
	Phase 3	4.3^a^ ± 1.58	3.7 ± 2.06	0.042
p*		<0.001	0.257	
Guilt	Phase 1	2.7^b^ ± 1.18	3.3 ± 2.65	0.009
	Phase2	4.1^a^ ± 1.26	3.7 ± 1.95	0.052
	Phase 3	4.2^a^ ± 1.38	3.7 ± 2.11	0.036
p*		<0.001	0.142	

#Independent group T-test *Repeated measures ANOVA test.

Statistically significant difference between groups in the same column is indicated by different lowercase letters.

^a, b:^Values with different superscript letters in the same row are significantly different (*p* < 0.05).

While the planning sub-factor score showed a significant difference in phase 1 (*p* = 0.004) and phase 2 (*p* = 0.015) according to BMI groups, it did not differ significantly in phase 3 (*p* > 0.05). While the phase 1 planning sub-factor score (mean = 3.236) was higher in overweight and obese women than in normal-weight women (mean = 2.661), the planning sub-factor score (mean = 4.177) of normal-weight women in phase 2 was higher than overweight and obese women (mean = 3.692). The positive reinforcement sub-factor score did not differ significantly in phase 1, phase 2 and phase 3 according to BMI groups (*p* > 0.05). In normal-weight women, the positive reinforcement sub-factor score is significantly higher in phase 1 than in phase 2 and phase 3. Negative reinforcement sub-factor scores differ significantly in phase 1 (*p* = 0.001), phase 2 (*p* = 0.011) and phase 3 (*p* = 0.034) according to BMI groups. In phase 1, the negative reinforcement sub-factor scores of overweight and obese women were higher than those of normal-weight women, while the negative reinforcement subscale scores of normal-weight women were higher than those of overweight and obese women in phase 2 and phase 3. In normal-weight women, the phase 2 and phase 3 negative reinforcement sub-factor scores were higher than the phase 1 negative reinforcement sub-factor score (*p* < 0.05). While the loss of control sub-factor scores differed significantly in phase 1 (*p* = 0.004) and phase 2 (*p* = 0.028) according to BMI groups, it did not differ significantly in phase 3 (*p* > 0.05). In phase 1, overweight and obese women had a higher loss of control sub-factor score (mean = 3.545) than women with normal weight (mean = 2.876). Food Concern and Hunger sub-factor scores differed significantly in phase 1 (*p* = 0.024) according to BMI groups but did not differ significantly in phase 2 and phase 3 (*p* > 0.05). In phase 1, overweight and obese women had higher Food Concern and Hunger sub-factor scores than normal-weight women. While the emotions sub-factor score differed significantly according to the BMI groups in phase 2 (*p* = 0.029) and phase 3 (*p* = 0.026), the emotions sub-factor scores in phase 1 was higher than those of overweight and obese women. The context sub-factor scores differ significantly in phase 1 (*p* = 0.001), phase 2 (*p* = 0.007) and phase 3 (*p* = 0.042) according to BMI groups. The Context sub-factor scores were higher in overweight and obese women than in normal-weight women in phase 1, while it was higher in normal-weight women than in obese women in phase 2 and phase 3. In normal-weight women, the phase 2 and phase 3. Context sub-factor scores were significantly higher than the phase 1 Context sub-factor scores (*p* < 0.05). While the guilt sub-factor score differed significantly in phase 1 (*p* = 0.00) and phase 3 (*p* = 0.036) according to BMI groups, it did not differ significantly in phase 2 (*p* > 0.05). In phase 1, the guilt sub-factor score was higher in overweight and obese women than in normal-weight women. In phase 3, it is higher in normal-weight women than in overweight and obese women (Table 3).

Table 4 displays the correlations between PFS total scores and dietary intake variables across menstrual cycle phases. In phase 1, significant positive correlations were found between PFS scores and energy intake, vegetable protein, and carbohydrate intake, suggesting that increased hedonic hunger during this phase may be associated with higher consumption of specific macronutrients. No statistically significant correlations were observed in Phases 2 and 3, indicating a potential phase-specific relationship between hedonic hunger and dietary intake.

**Table 4.** The correlation between energy and nutrient intake and PFS total score according to menstrual cycle phases

Energy and nutrients	PFS total score		
	Phase 1	Phase 2	Phase 3
Energy	0.202*	0.116	0.130
Protein	0.073	0.079	0.151
Vegetable protein	0.203*	0.024	0.152
Carbohydrate	0.193*	0.114	0.108
Fibre	0.034	0.028	0.090
Fat	0.135	0.074	0.083
Saturated fat	0.126	0.147	0.025
MUFA	0.130	0.010	0.112
PUFA	0.080	0.021	0.093
Omega-3	0.050	0.087	0.013
Omega-6	0.068	0.008	0.120
Cholesterol	-0.035	0.148	0.072
Sodium	0.019	-0.022	-0.045

Pearson correlation.

A positive correlation was found between women’s total PFS score and energy, vegetable protein, and carbohydrate intake in phase 1. Correlation relationships between other variables are not statistically significant (*p* > 0.05) (Table 4).

Table 5 presents the PFS total and sub-factor scores across menstrual cycle phases, stratified by the presence or absence of PMS. Across all three phases, women with PMS consistently showed significantly higher PFS total and sub-factor scores compared to those without PMS. This suggests that the presence of PMS is associated with elevated hedonic hunger and heightened responsiveness to food cues throughout the menstrual cycle.

**Table 5.** Evaluation of PFS total and sub-factor scores according to menstrual cycle phases and PMS status

PFS	Phase 1			Phase 2			Phase 3		
	PMS			PMS			PMS		
	Yes (*n* = 104)	No (*n* = 46)		Yes (*n* = 104)	No (*n* = 46)		Yes (*n* = 104)	No (*n* = 46)	
	Means ± SD	Means ± SD	*p* ^#^	Means ± SD	Means ± SD	*p* ^#^	Means ± SD	Means ± SD	*p* ^#^
PFS total score	3.2 ± 1.00	2.7 ± 0.81	0.003	3.2 ± 0.94	2.7 ± 0.94	0.004	3.2 ± 0.94	2.6 ± 0.89	<0.001
Food available	2.8 ± 1.01	2.4 ± 0.93	0.031	2.8 ± 1.03	2.4 ± 1.06	0.051	2.9 ± 1.07	2.4 ± 1.01	0.010
Food present	3.4 ± 1.11	2.8 ± 0.89	0.002	3.3 ± 1.06	2.7 ± 0.96	0.001	3.4 ± 0.99	2.7 ± 0.97	<0.001
Food tasted	3.5 ± 1.12	2.9 ± 0.93	0.010	3.5 ± 1.04	2.9 ± 1.06	0.003	3.5 ± 1.10	2.8 ± 0.99	<0.001

#Independent groups T-test.

**Table 6.** Linear regression analysis for hedonic hunger prediction

	PFS total score (Phase 1)			PFS total score (Phase 2)			PFS total score (Phase 3)		
Model	Beta	*t*	*p*-value	Beta	*t*	*p*-value	Beta	*t*	*p*-value
PMS	–0.223	–2.781	0.006*	–0.237	–2.965	0.004*	–0.285	–3.615	0.000*
BMI	0.205	2.546	0.012*	0.238	2.982	0.003*	0.151	1.862	0.065

According to the presence of PMS, phase 1, phase 2 and phase 3 PFS total and sub-factor scores of women differ significantly (*p* < 0.05). The scores of those with PMS were higher than the scores of those without PMS (Table 5).

## Discussion

The change in the food consumption status of women according to the menstrual cycle have been studied for many years in the literature. According to these studies, energy intake seems to be higher in the luteal phase.^(25–28,30)^ However, there is not enough information about the change of hedonic hunger and excessive food desire according to menstrual cycle phases.^(2)^ In addition, the presence of PMS can affect eating behaviour.^(6,7,43)^ Therefore, in this study, we aimed to evaluate hedonic hunger and food cravings according to the presence of PMS and menstrual cycle phases. The main findings of our study are; (i) individuals with PMS have higher PFS total scores than individuals without PMS in all of the menstrual cycle phases, (ii) overweight and obese individuals have higher PFS total, food available and food present sub-factor scores in menstrual cycle phases 1 and 2 than normal individuals, (iii) PFS scores of individuals did not differ according to menstrual cycle phases.

PMS is a condition that affects a substantial proportion of women of reproductive age. In individuals with PMS, eating behaviors may be influenced by the cyclical physical and emotional symptoms associated with the luteal phase.”^(44)^ Consumption of high energy, fat, simple sugar and salty foods has been shown to be associated with the presence of PMS.^(44)^ Previous studies have shown that adherence to a Western-style diet — characterized by high intake of refined carbohydrates, saturated fats, and processed foods — is associated with a higher prevalence and severity of PMS symptoms. However, it remains unclear whether this dietary pattern contributes to PMS or if individuals experiencing PMS symptoms are more likely to adopt such eating behaviours.^(45,46)^ In a study examining the relationship between adherence to a Mediterranean diet and the presence of PMS, it was shown that individuals with PMS had a lower Mediterranean diet score.^(47)^ However, to our knowledge, there is no study examining the relationship between PMS and hedonic hunger and food craving. In this study, we evaluated the hedonic hunger and excessive food cravings of young adult women of childbearing age according to their PMS status and observed that the presence of PMS increased hedonic hunger during the menstrual cycle phases, as indicated by our regression model (Table 6).

Fluctuations in the levels of ovarian hormones during the menstrual cycle phases may cause differences in appetite. Among the main phases of the menstrual cycle, follicular and luteal phases, high oestrogen and low progesterone levels are observed in the follicular phase, while high progesterone and moderate oestrogen levels are observed in the luteal phase. It is suggested that the decrease in oestrogen levels during the luteal phase is a factor that increases homeostatic and hedonic appetite.^(2)^ In addition, Lima et al. observed that women with PMS reported significantly higher levels of hedonic appetite across the menstrual cycle, particularly in phases characterized by hormonal fluctuation, such as the late luteal and early follicular phases. These patterns were linked to mood changes and emotional dysregulation, reinforcing the role of serotonin and progesterone in modulating appetite responses.^(2)^ These findings are in line with neuroimaging evidence indicating that sex hormones, particularly progesterone, influence the activation of homeostatic, emotional, and attentional brain regions in response to food cues. Such hormonal modulation across the menstrual cycle may partly explain variations in eating behaviour and hedonic food responses observed in women, especially during the luteal phase.^(16)^ In support of this suggestion, according to our results, food craving sub-factors differ significantly between follicular and ovulation phases in normal-weight individuals. However, no significant difference was found in PFS scores. The fact that the questions of the PFS used to measure hedonic hunger are more general may explain the interphase insignificance.

Strengths of the study include its longitudinal, repeated measures design, which allowed us to assess changes in hedonic hunger and food craving across three distinct phases of the menstrual cycle within the same participants. This design reduces inter-individual variability and enhances the reliability of within-subject comparisons. Additionally, the relatively large and homogeneous sample provides subgroup analyses based on PMS status and BMI classification. The application of validated tools (PFS and FCQ-T) across hormonally dynamic phases contributes valuable insight into the behavioural effects of menstrual cycle variations. This study has several limitations that should be acknowledged. First limitation of this study is that PMS was classified based on a single self-report questionnaire. While the PMSS is a validated tool, the gold standard for PMS diagnosis involves prospective daily symptom ratings across two or more menstrual cycles, Therefore, the PMS classification in this study may be subject to recall bias and may not fully reflect the cyclical nature of the symptoms. Second, menstrual cycle phases were determined based on self-reported cycle dates without hormonal validation, which may have introduced misclassification. Third, all anthropometric and dietary data were self-reported, which may be subject to recall or reporting bias. Fourth, while the FCQ-T is designed as a trait-based measure, it was administered across multiple cycle phases to explore possible variation in craving tendencies; this approach, though supported by recent literature, may not fully capture state-level fluctuations. Additionally, our sample consisted solely of women with regular menstrual cycles and without major chronic or psychiatric conditions, which may limit generalizability. Although data were collected over the course of a single menstrual cycle (approximately one month), this duration was chosen to capture variations across distinct hormonal phases. However, we acknowledge that a longer observation period could provide more robust insights into cyclical patterns and inter-cycle variability. Future studies should consider extended follow-up periods to enhance the generalizability of findings. Finally, cultural and regional dietary patterns may also influence the findings and should be considered in future cross-cultural studies.

## Conclusion

In conclusion, the presence of PMS significantly affects hedonic eating behaviour across menstrual cycle phases, and BMI was also found to be an influential factor, particularly in phase 1 and phase 2. These findings suggest that individualized dietary counseling and behavioural interventions tailored to menstrual cycle phases and PMS status may be beneficial for women at risk of disordered eating patterns. Future studies should consider including hormonal assays to more precisely characterize cycle phases, use larger and more diverse populations to enhance generalizability, and apply objective methods for dietary tracking to reduce self-report bias. A longitudinal or interventional design could also provide more definitive insights into causality and the effectiveness of targeted strategies.

## Data Availability

The datasets used and/or analysed during the current study are available from the corresponding author on reasonable request.
